# Inflammatory Monocytes and Neutrophils Regulate Streptococcus suis-Induced Systemic Inflammation and Disease but Are Not Critical for the Development of Central Nervous System Disease in a Mouse Model of Infection

**DOI:** 10.1128/IAI.00787-19

**Published:** 2020-02-20

**Authors:** Jean-Philippe Auger, Serge Rivest, Marie-Odile Benoit-Biancamano, Mariela Segura, Marcelo Gottschalk

**Affiliations:** a Research Group on Infectious Diseases in Production Animals, University of Montreal, Saint-Hyacinthe, Quebec, Canada; b Swine and Poultry Infectious Diseases Research Center, University of Montreal, Saint-Hyacinthe, Quebec, Canada; c Department of Pathology and Microbiology, Faculty of Veterinary Medicine, University of Montreal, Saint-Hyacinthe, Quebec, Canada; d CHU de Québec Research Center and Department of Molecular Medicine, Faculty of Medicine, Laval University, Quebec, Quebec, Canada; Washington State University

**Keywords:** *Streptococcus suis*, inflammation, meningitis, monocytes, neutrophils, sepsis

## Abstract

Streptococcus suis is an important porcine bacterial pathogen and zoonotic agent responsible for sudden death, septic shock, and meningitis. These pathologies are a consequence of elevated bacterial replication leading to exacerbated and uncontrolled inflammation, a hallmark of the S. suis systemic and central nervous system (CNS) infections. Monocytes and neutrophils are immune cells involved in various functions, including proinflammatory mediator production. Moreover, monocytes are composed of two main subsets: shorter-lived inflammatory monocytes and longer-lived patrolling monocytes. However, regardless of their presence in blood and the fact that S. suis-induced meningitis is characterized by infiltration of monocytes and neutrophils into the CNS, their role during the S. suis systemic and CNS diseases remains unknown. Consequently, we hypothesized that monocytes and neutrophils participate in S. suis infection via bacterial clearance and inflammation. Results demonstrated that inflammatory monocytes and neutrophils regulate S. suis-induced systemic disease via their role in inflammation required for bacterial burden control. In the CNS, inflammatory monocytes contributed to exacerbation of S. suis-induced local inflammation, while neutrophils participated in bacterial burden control. However, development of clinical CNS disease was independent of both cell types, indicating that resident immune cells are mostly responsible for S. suis-induced CNS inflammation and clinical disease and that inflammatory monocyte and neutrophil infiltration is a consequence of the induced inflammation. In contrast, the implication of patrolling monocytes was minimal throughout the S. suis infection. Consequently, this study demonstrates that while inflammatory monocytes and neutrophils modulate S. suis-induced systemic inflammation and disease, they are not critical for CNS disease development.

## Abstract (precis)

Streptococcus suis is an important porcine bacterial pathogen and zoonotic agent responsible for sudden death, septic shock, and meningitis. These pathologies are a consequence of elevated bacterial replication leading to exacerbated and uncontrolled inflammation, a hallmark of the S. suis systemic and central nervous system (CNS) infections. Monocytes and neutrophils are immune cells involved in various functions, including proinflammatory mediator production.

## INTRODUCTION

Streptococcus suis is one of the most important porcine bacterial pathogens and is a zoonotic agent mainly responsible for sudden death (pigs), septic shock (humans), and meningitis (both species) (1, 2). These pathologies are characterized by an exacerbated and uncontrolled inflammation, which is a hallmark of the S. suis systemic and central nervous system (CNS) infections (3, 4). Of the different serotypes described, serotype 2 is the most virulent and widespread worldwide (5).

Following colonization of the upper respiratory tract of pigs, S. suis may reach the bloodstream by breaching the mucosa or via other poorly understood mechanisms (6). Infection of humans occurs via skin wounds or at the intestinal interface following contact with diseased animals and/or raw or undercooked infected pork products (6). In the bloodstream, S. suis resists killing by phagocytes, which results in bacteremia, organ dissemination and development of systemic infection (7). Moreover, activation of innate immune cells leads to an exacerbated inflammation responsible for sepsis leading to sudden death in pigs and septic shock in humans (1). If untreated, S. suis-induced systemic inflammation may result in host death (1). Moreover, if bacteria are not rapidly cleared from the bloodstream, they can reach the blood-brain barrier or blood-cerebrospinal fluid barrier, which they can then cross (7). However, the events leading to the development of CNS disease, which is characterized by meningitis, remain misunderstood (7). Though meningitis is associated with an excessive local inflammation and infiltration of monocytes and neutrophils (3, 8, 9), it is unknown whether these cells are a cause or a consequence of this inflammatory response.

The interactions between S. suis and innate immune cells, particularly phagocytes, have been somewhat dissected, with studies mostly focusing on macrophages and conventional dendritic cells (DCs), which are mainly tissue-resident cells (10–17). However, not only are monocytes and neutrophils the main phagocytes in blood (18), but they are also sources of proinflammatory mediators and play important roles during bacterial infection (19, 20). Moreover, S. suis-induced meningitis is characterized by an infiltration of these cells into the CNS (2, 3, 8, 21). However, their role during S. suis-induced systemic and CNS diseases has been little studied. In fact, information is limited to only a few *in vitro* studies (22–25).

Though historically viewed as only being precursor cells responsible for replenishing tissue-resident macrophage and DC populations, monocytes are mature effector cells involved in a variety of functions, of which phagocytosis and proinflammatory mediator production are the most important (26). They are composed of morphologically and phenotypically heterogeneous subsets (with different roles), which are similar between humans, pigs, and mice (26). The two main subsets are the shorter-lived “classical” inflammatory monocytes (Ly6C^hi^CCR2^hi^CX_3_CR1^lo^ in mice) that infiltrate inflamed tissues to trigger local immune responses and the longer-lived “nonclassical” patrolling monocytes (Ly6C^lo^CCR2^lo^CX_3_CR1^hi^ in mice) that home in to noninflamed tissues and repopulate tissue-resident cells during homeostatic conditions (27). While egress of inflammatory monocytes from the bone marrow and their mobilization require C-C chemokine receptor (CCR) type 2 (CCR2), differentiation and survival of patrolling monocytes depend on the transcription factor nuclear receptor subfamily 4 group A member 1 (Nr4a1) (28, 29). More recently, however, their roles have become much less clearly defined. Indeed, though they are fully differentiated upon exiting the bone marrow, current research suggests that monocytes can shift between subsets in peripheral blood (26, 30). Whereas the role of inflammatory monocytes depends on the pathogen, being beneficial during Listeria monocytogenes and Escherichia coli K1 infections but playing no role during Streptococcus pneumoniae meningitis, that of patrolling monocytes during bacterial infection remains virtually unknown (28, 31, 32).

On the other hand, neutrophils represent the most abundant innate immune cells in blood (18). As such, they play important roles in bacterial clearance and immune responses, including phagocytosis and killing, degranulation, neutrophil extracellular trap formation, and proinflammatory mediator production (20). Moreover, neutrophils migrate to infected tissues, where their presence is often decisive to the outcome. Indeed, neutrophils play a beneficial role during group B *Streptococcus* (GBS) and S. pneumoniae infections via their participation in inflammation required for bacterial control and clearance (31, 33).

To further our knowledge of the S. suis pathogenesis, the role of inflammatory and patrolling monocytes, as well as neutrophils, during systemic and CNS infections was evaluated. We demonstrated that inflammatory monocytes and neutrophils, but not patrolling monocytes, help control S. suis-induced systemic disease via their role in inflammation, which subsequently controls bacterial burden. Meanwhile, inflammatory monocytes contribute to the exacerbation of S. suis-induced CNS inflammation, while neutrophils participate in CNS bacterial burden control, although development of clinical CNS disease is independent of both cell types. Therefore, inflammatory monocytes and neutrophils have differential roles during the S. suis-induced systemic and CNS diseases. Finally, the nonredundant role of inflammatory monocytes and neutrophils in the CNS indicates that resident immune cells are mostly responsible for CNS inflammation and clinical disease and that their infiltration is a consequence of the induced inflammatory response.

## RESULTS

### Inflammatory but not patrolling monocytes are implicated in host survival during S. suis systemic infection.

**FIG 1:**
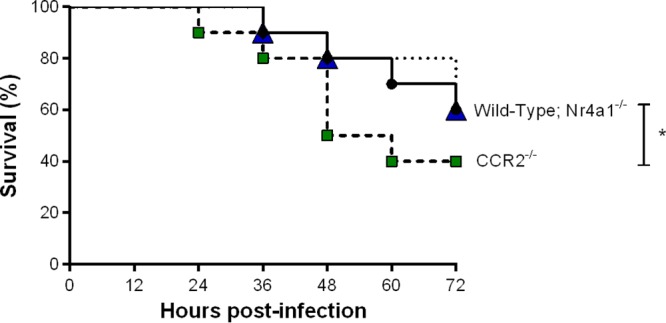
Inflammatory but not patrolling monocytes are implicated in host survival during S. suis systemic infection. Survival of wild-type (black), CCR2^−/−^ (green), and Nr4a1^−/−^ (blue) mice infected with S. suis by intraperitoneal inoculation during the acute systemic infection (until 72 h postinfection). The data represent survival curves (*n* = 10). * (*P* < 0.05) indicates a significant difference between survival of wild-type and CCR2^−/−^ mice as determined using the log-rank (Mantel-Cox) test.

Previous studies suggested that monocytes interact with S. suis (22, 24, 34). However, these studies were conducted *in vitro* and have not evaluated the contribution of monocyte subsets. Consequently, the role of inflammatory and patrolling monocytes was evaluated during the acute S. suis systemic infection following intraperitoneal inoculation of a standard dose of 1 × 10^7^ CFU using CCR2^−/−^ and Nr4a1^−/−^ mice, which are required for inflammatory and patrolling monocyte mobilization and survival, respectively (28, 29). Following the acute systemic disease (72 h postinfection), survival of CCR2^−/−^ mice was significantly less than that of wild-type and Nr4a1^−/−^ mice (*P* < 0.05), whose survival was similar (Fig. 1).

**FIG 2:**
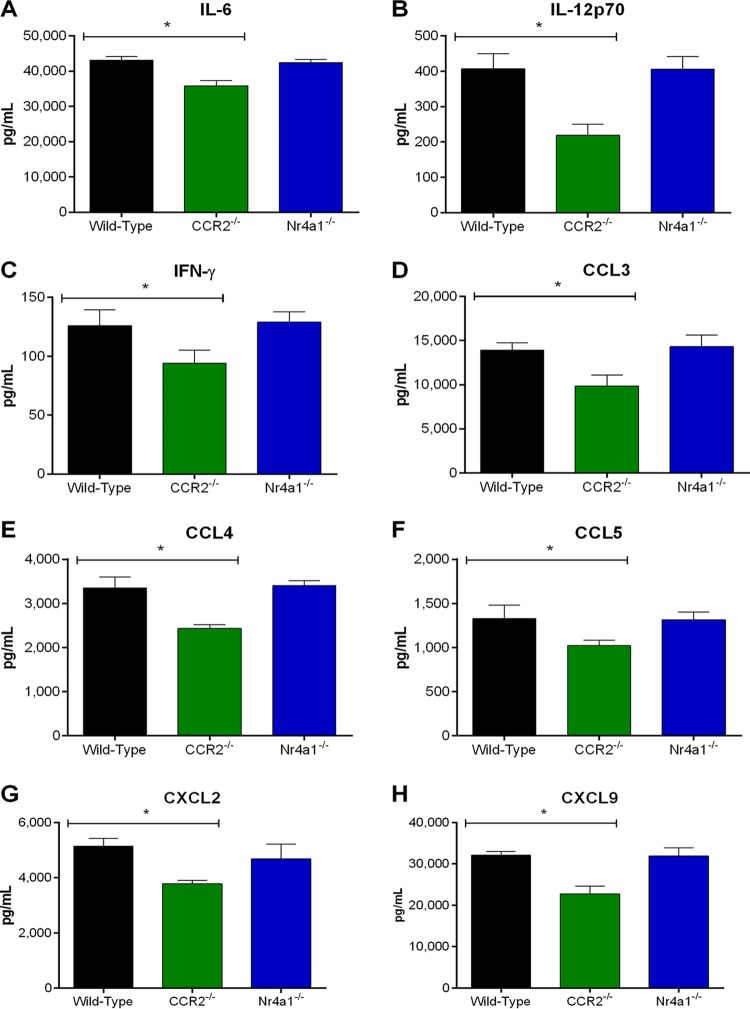
Inflammatory but not patrolling monocytes contribute to plasma proinflammatory mediator production involved in S. suis-induced systemic inflammation. Plasma levels of IL-6 (A), IL-12p70 (B), IFN-γ (C), CCL3 (D), CCL4 (E), CCL5 (F), CXCL2 (G), and CXCL9 (H) in wild-type, CCR2^−/−^, and Nr4a1^−/−^ mice 12 h following infection with S. suis by intraperitoneal inoculation. The data represent the mean ± SEM (*n* = 8). * (*P* < 0.05) indicates a significant difference in plasma levels between wild-type and CCR2^−/−^ mice as determined using the unpaired *t* test.

Though host death during the S. suis systemic infection is usually due to an exacerbated inflammatory response, a certain level of inflammation is required for bacterial elimination (10). As such, the role of monocytes in systemic inflammation was evaluated by measuring plasma mediators from wild-type, CCR2^−/−^, and Nr4a1^−/−^ mice 12 h postinfection, corresponding to the time when production is maximal (4, 35). As previously described, S. suis induced high plasmatic levels of the different proinflammatory mediators measured (interleukin-6 [IL-6], IL-12p70, interferon-γ [IFN-γ], C-C motif chemokine ligand 3 [CCL3], CCL4, CCL5, C-X-C motif chemokine ligand 2 [CXCL2], and CXCL9) in wild-type mice (Fig. 2) (4, 35). Levels of these mediators in the plasma of Nr4a1^−/−^ mice were similar to those of wild-type mice (Fig. 2). In contrast, systemic levels of proinflammatory mediators were significantly lower in CCR2^−/−^ mice (*P* < 0.05), with a 20% reduction in IL-6 and IFN-γ production, a 30% to 40% reduction in chemokine production (CCL3, CCL4, CCL5, CXCL2, and CXCL9), and a 50% reduction in IL-12p70 production (Fig. 2).

**FIG 3:**
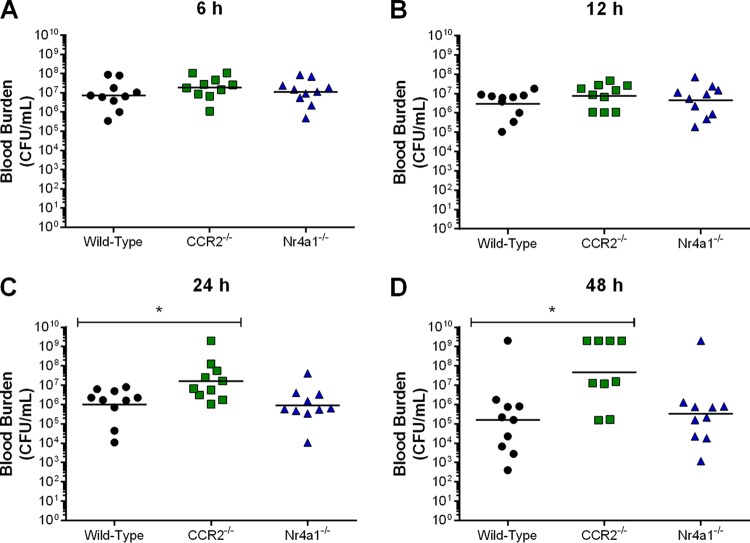
Inflammatory but not patrolling monocytes participate in blood bacterial burden control following S. suis infection. Blood bacterial burden of wild-type, CCR2^−/−^, and Nr4a1^−/−^ mice 6 h (A), 12 h (B), 24 h (C), and 48 h (D) following infection with S. suis by intraperitoneal inoculation. The data represent the geometric mean (*n* = 10). A blood bacterial burden of 2 × 10^9^ CFU/ml, corresponding to the average burden upon euthanasia, was attributed to euthanized mice. * (*P* < 0.05) indicates a significant difference between the blood bacterial burden of wild-type and CCR2^−/−^ mice as determined using the Mann-Whitney rank sum test.

Given these differences in systemic inflammation and because inflammation participates in bacterial clearance, bacteremia of wild-type, CCR2^−/−^, and Nr4a1^−/−^ mice was evaluated at different times following infection. No differences were observed in blood bacterial burden early after infection (6 h and 12 h postinfection) (Fig. 3A and B). However, since inflammation must first be induced to have an effect, this was not entirely surprising. At later times (24 h and 48 h postinfection), however, blood bacterial burden was significantly greater in CCR2^−/−^ mice than in wild-type and Nr4a1^−/−^ mice (*P* < 0.01) (Fig. 3C and D). This suggests that inflammatory monocytes are indirectly implicated in blood bacterial burden control via modulation of inflammation.

### Neutrophils are required for host survival during S. suis systemic infection.

**FIG 4:**
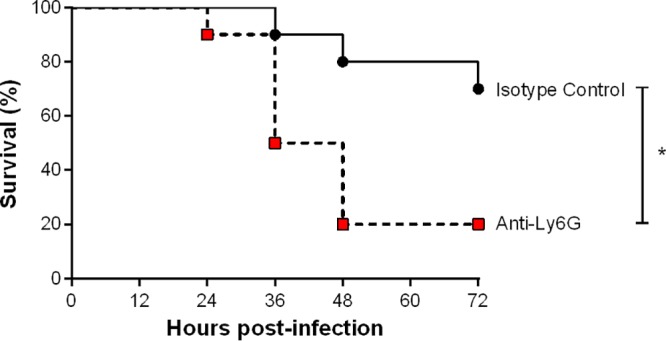
Neutrophils are implicated in host survival during S. suis systemic infection. Survival of wild-type mice pretreated with either isotype control (black) or anti-Ly6G neutralizing antibody (red) infected with S. suis by intraperitoneal inoculation during the acute systemic infection (until 72 h postinfection). The data represent survival curves (*n* = 10). * (*P* < 0.05) indicates a significant difference between the survival of isotype control- and anti-Ly6G-treated mice as determined using the log-rank (Mantel-Cox) test.

The partial contribution of monocytes in host survival during S. suis systemic infection suggested that other innate immune cells are also involved. Since neutrophils are the most abundant innate immune cells in blood, their role was evaluated. Neutrophils were depleted from wild-type mice by injection of the 1A8 anti-Ly6G neutralizing antibody or its isotype control 24 h prior to infection with a standard dose of 1 × 10^7^ CFU of S. suis. Treatment depleted neutrophils beyond 85%, with low counts persisting for at least 72 h (see Table S1 in the supplemental material). Depletion of neutrophils resulted in significantly less survival during the acute systemic infection (until 72 h postinfection), with only 20% of mice remaining alive by 48 h postinfection (*P* < 0.01) (Fig. 4).

**FIG 5:**
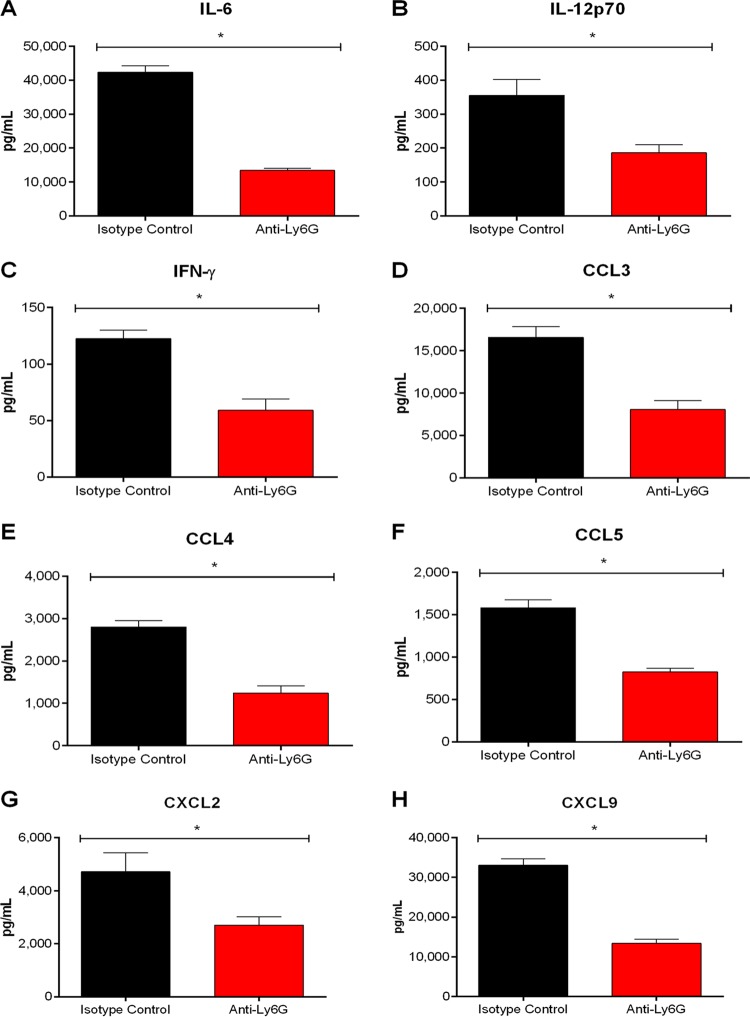
Neutrophils contribute to plasma proinflammatory mediator production involved in S. suis-induced systemic inflammation. Plasma levels of IL-6 (A), IL-12p70 (B), IFN-γ (C), CCL3 (D), CCL4 (E), CCL5 (F), CXCL2 (G), and CXCL9 (H) in wild-type mice pretreated with either isotype control or anti-Ly6G neutralizing antibody 12 h following infection with S. suis by intraperitoneal inoculation. The data represent the mean ± SEM (*n* = 8). * (*P* < 0.05) indicates a significant difference in plasma levels between isotype control- and anti-Ly6G-treated mice as determined using the unpaired *t* test.

**FIG 6:**
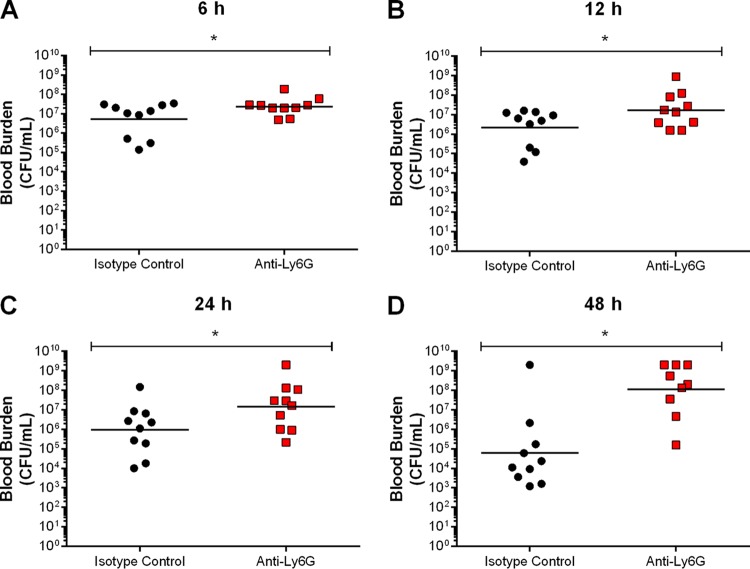
Neutrophils participate in blood bacterial burden control following S. suis infection. Blood bacterial burden of wild-type mice pretreated with either isotype control or anti-Ly6G neutralizing antibody 6 h (A), 12 h (B), 24 h (C), and 48 h (D) following infection with S. suis by intraperitoneal inoculation. The data represent the geometric mean (*n* = 10). A blood bacterial burden of 2 × 10^9^ CFU/ml, corresponding to the average burden upon euthanasia, was attributed to euthanized mice. * (*P* < 0.05) indicates a significant difference between the blood bacterial burden of isotype control- and anti-Ly6G-treated mice as determined using the Mann-Whitney rank sum test.

To better explain this difference in survival, the role of neutrophils in inflammation was evaluated by measuring plasma mediators 12 h postinfection. Neutrophil depletion resulted in a significant reduction of all systemic proinflammatory mediators evaluated, equivalent to a 50% to 60% decrease, and a notable 75% decrease for IL-6 production (*P* < 0.01) (Fig. 5). Given this important modulation of systemic inflammation by neutrophils, their effect on blood bacterial burden was determined. Accordingly, depletion of neutrophils resulted in significantly greater bacteremia as of 6 h postinfection (*P* < 0.01) (Fig. 6), which is prior to maximum inflammatory production (35). This rapid increase in burden following depletion suggests that neutrophils are probably implicated in blood bacterial burden control via modulation of inflammation and direct killing mechanisms.

### S. suis induces a rapid, massive, and time-dependent release of proinflammatory chemokines from the CNS.

**FIG 7:**
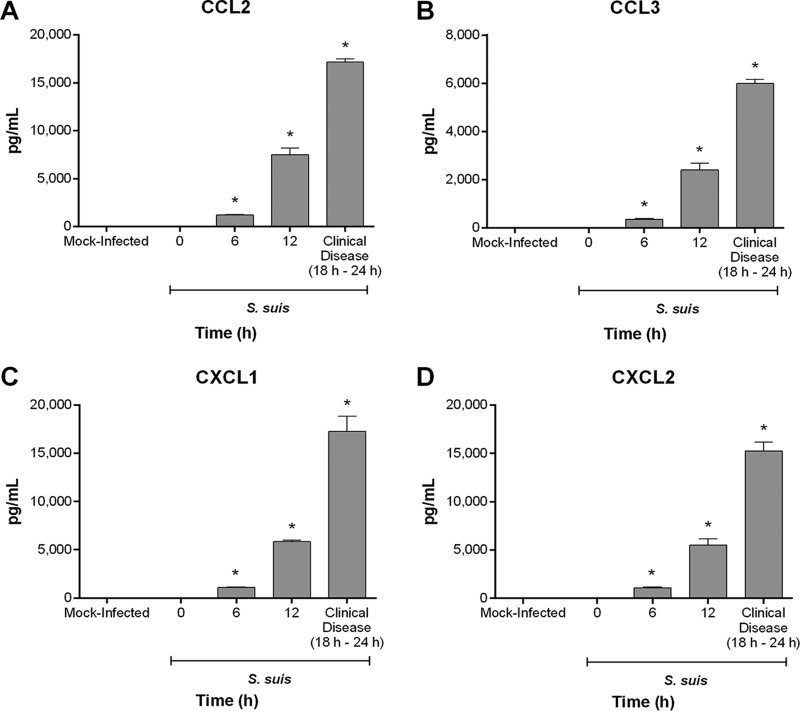
S. suis induces a rapid, massive, and time-dependent release of proinflammatory chemokines from the central nervous system. Brain levels of CCL2 (A), CCL3 (B), CXCL1 (C), and CXCL2 (D) in wild-type mice at different times following mock infection (THB) or intracisternal infection with 10^5^ CFU of S. suis. The data represent the mean ± SEM (*n* = 5). * (*P* < 0.05) indicates a significant difference in production with 0 h as determined using the unpaired *t* test.

Following the acute S. suis systemic infection, surviving individuals are susceptible to developing a CNS disease of which meningitis is the hallmark (3, 4). This disease is characterized by an exacerbated local inflammatory response in the CNS, activation of local resident CNS immune cells, and infiltration of peripheral immune cells, namely, monocytes and neutrophils (9, 21, 35). Moreover, this inflammatory response is composed not only of proinflammatory cytokines, but also of chemokines, which might participate in the recruitment and/or amplification of monocytes and neutrophils (4). However, production of these chemokines has always been evaluated upon presentation of clinical CNS disease, making it difficult to understand the kinetics. Consequently, production of CCL2, CCL3, CXCL1, and CXCL2, important for the chemoattraction of monocytes and neutrophils, was evaluated at different times following inoculation of S. suis via the transcutaneal intracisternal route, as this well-developed model results in the rapid development of clinical signs and histopathological lesions of CNS disease (4, 36). While chemokine levels were undetectable in mock-infected mice regardless of time, levels of CCL2, CCL3, CXCL1, and CXCL2 were not only significantly higher 6 h postinfection (*P* < 0.01), but were massive in themselves (Fig. 7). Levels increased with time before reaching their maximum upon presentation of clinical disease (between 18 h and 24 h postinfection) (Fig. 7). Consequently, S. suis induces a rapid, massive, and time-dependent release of proinflammatory chemokines from the CNS.

### Inflammatory monocytes and neutrophils infiltrate the brain during S. suis-induced CNS disease.

**FIG 8:**
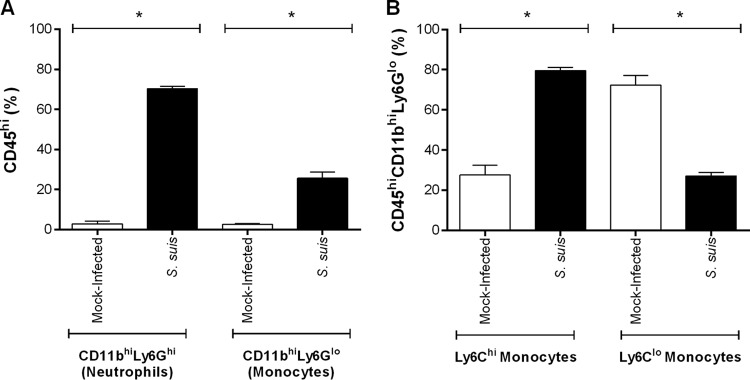
S. suis induces the infiltration of neutrophils, and to a lesser extent of inflammatory monocytes, into the central nervous system (CNS) following infection. Infiltrating CD45^hi^CD11b^hi^Ly6G^hi^ neutrophils and CD45^hi^CD11b^hi^Ly6G^lo^ monocytes in the CNS of mock-infected and S. suis-infected mice (upon presentation of clinical CNS disease) were analyzed using flow cytometry (A). Inflammatory (Ly6C^hi^) and patrolling (Ly6C^lo^) monocyte subpopulations (B) were determined by gating on the CD45^hi^CD11b^hi^Ly6G^lo^ monocytes in panel A. The data represent the mean ± SEM (*n* = 3). * (*P* < 0.05) indicates a significant difference between mock-infected and S. suis-infected mice as determined using the Mann-Whitney rank sum test.

Though monocytes and neutrophils have been observed in CNS lesions of animals following S. suis infection using histology (2, 3, 8, 21), the proportions of these cell types have not been determined before. Consequently, infiltrating brain monocyte and neutrophil populations (CD45^hi^CD11b^hi^) were quantified by flow cytometry upon presentation of S. suis-induced clinical CNS disease in wild-type mice. Neutrophils and monocytes were distinguished based on their Ly6G expression, with Ly6G^hi^ and Ly6G^lo^ populations corresponding to neutrophils and monocytes, respectively. In mock-infected mice, neutrophils and monocytes were rare, with less than 3% (Fig. 8A). Meanwhile, S. suis CNS infection induced a significant increase, with 70% for neutrophils and 30% for monocytes (*P* > 0.05) (Fig. 8A). Furthermore, of the rare monocytes present in the CNS of mock-infected mice, 70% were Ly6C^lo^, corresponding to patrolling monocytes (Fig. 8B). Interestingly, S. suis infection greatly altered this ratio, with infiltrating monocytes being composed of 80% Ly6C^hi^ inflammatory monocytes and only 20% Ly6C^lo^ patrolling monocytes (Fig. 8B).

### Differential role of infiltrating monocytes and neutrophils during S. suis-induced CNS disease.

**FIG 9:**
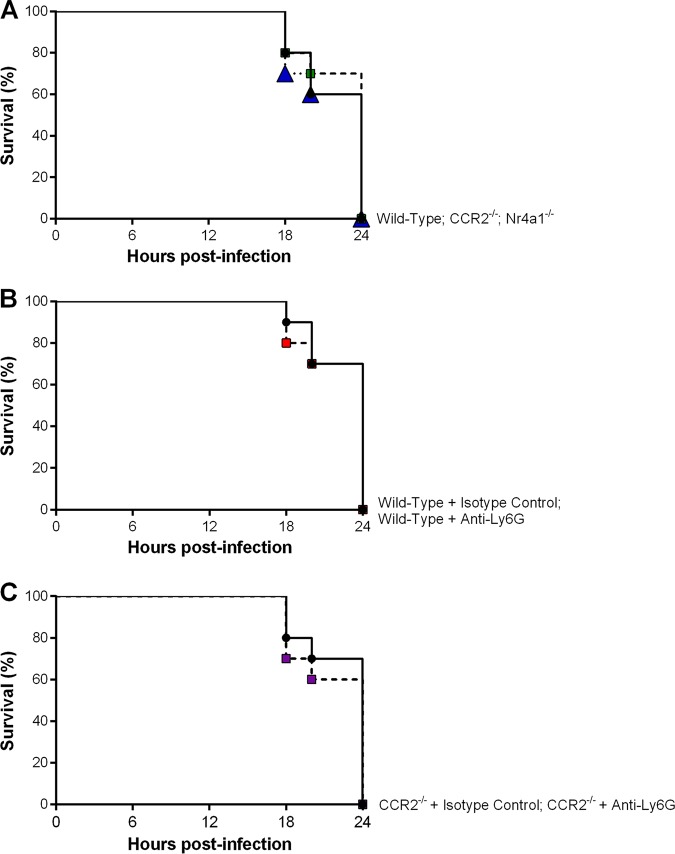
Monocytes and neutrophils are not required for the development of clinical central nervous system (CNS) disease following S. suis infection. Survival of wild-type (black), CCR2^−/−^ (green), and Nr4a1^−/−^ (blue) mice (A), wild-type mice pretreated with either isotype control (black) or anti-Ly6G neutralizing antibody (red) (B), and CCR2^−/−^ mice pretreated with either isotype control (black) or anti-Ly6G neutralizing antibody (purple) (C) following intracisternal infection with 10^5^ CFU of S. suis. The data represent the survival curves of mice euthanized upon presentation of clinical signs of CNS disease (*n* = 10). Statistical differences were analyzed using the log-rank (Mantel-Cox) test.

Given the monocyte and neutrophil infiltrates observed in the CNS of S. suis-infected animals upon presentation of clinical disease, the role of infiltrating monocytes and neutrophils in the development of S. suis-induced CNS disease was evaluated. Wild-type, CCR2^−/−^, and Nr4a1^−/−^ mice (monocytes) or wild-type mice treated with anti-Ly6G (neutrophils) or the isotype control were infected with a standard dose (1 × 10^5^ CFU) of S. suis using the transcutaneal intracisternal route, and the development of clinical signs of CNS disease was evaluated. Since mice were euthanized upon presentation of clinical signs, data are presented as survival curves. Of the wild-type, CCR2^−/−^, and Nr4a1^−/−^ mice infected with S. suis, 100% developed clinical signs of CNS disease within 24 h postinfection (Fig. 9A). Similar results were obtained following neutrophil depletion, with 100% of isotype control- or anti-Ly6G-treated mice developing clinical signs of CNS disease within 24 h of S. suis infection (Fig. 9B). In contrast, none of the mock-infected mice developed clinical signs of infection (data not shown). These results were confirmed by histopathology, with S. suis-infected mice presenting classical lesions of CNS infection, including massive suppuration, leukocyte infiltration, multifocal gliosis, hemorrhages, and necrosis, indicating important CNS tissue damage and inflammation (Fig. S2 and S3). Meanwhile, no histopathological lesions were observed in mock-infected mice (Fig. S2 and S3). Importantly, infiltration of inflammatory monocytes and neutrophils into the CNS of S. suis-infected mice upon presentation of clinical disease was reduced by more than 80% in CCR2^−/−^ and anti-Ly6G-treated mice, respectively, in comparison to wild-type or isotype-treated mice (data not shown).

Given the lack of a role of infiltrating inflammatory monocytes and neutrophils in the development of clinical S. suis-induced CNS disease, it was hypothesized that the presence of the other cell types might compensate, given the susceptibility of the CNS to S. suis infection (4). Consequently, CCR2^−/−^ mice were treated with the anti-Ly6G neutralizing antibody (or isotype control) as described above in order to deplete neutrophils in the absence of inflammatory monocytes. Nevertheless, 100% of isotype control- and anti-Ly6G-treated CCR2^−/−^ mice developed clinical signs of CNS disease within 24 h of S. suis infection, similarly to what occurred in the absence of inflammatory monocytes or neutrophils individually (Fig. 9C), which was also confirmed by histopathology, where lesions of CNS tissue damage and inflammation were observed (Fig. S4).

**FIG 10:**
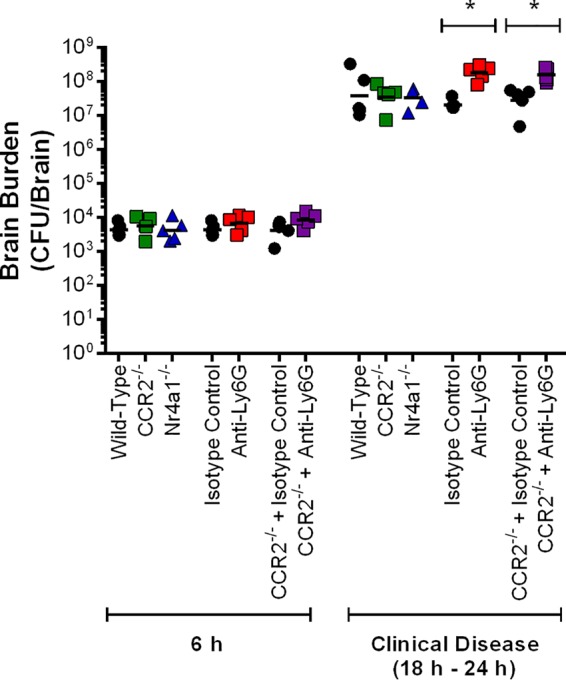
Neutrophils but not monocytes participate in brain bacterial burden control following S. suis infection. Brain bacterial burden of wild-type, CCR2^−/−^, and Nr4a1^−/−^ mice, wild-type mice pretreated with either isotype control or anti-Ly6G neutralizing antibody, and CCR2^−/−^ mice pretreated with either isotype control or anti-Ly6G neutralizing antibody 6 h following intracisternal infection with 10^5^ CFU of S. suis or upon presentation of clinical central nervous system disease. The data represent the geometric mean (*n* = 5). * (*P* < 0.05) indicates a significant difference between the blood bacterial burden of isotype control- and anti-Ly6G-treated mice (wild-type or CCR2^−/−^) as determined using the Mann-Whitney rank sum test.

The development of S. suis-induced CNS disease is usually due to bacterial presence and replication in the CNS, which lead to inflammation (4). As such, brain bacterial loads were evaluated early following infection (6 h) and upon presentation of clinical signs of S. suis-induced CNS disease (between 18 h and 24 h postinfection). Loads were similar between wild-type, CCR2^−/−^, and Nr4a1^−/−^ mice 6 h postinfection, averaging 5 × 10^3^ CFU (Fig. 10). Furthermore, no differences were observed upon presentation of clinical CNS disease (Fig. 10). While depletion of neutrophils had no effect on brain bacterial loads 6 h postinfection, they were significantly greater in neutrophil-depleted mice upon presentation of clinical signs of CNS disease (*P* < 0.05), regardless of similar susceptibility to the infection (Fig. 10). Brain bacterial loads of anti-Ly6G-treated CCR2^−/−^ mice were similar to those of anti-Ly6G-treated wild-type mice both at 6 h postinfection and upon presentation of clinical CNS disease (Fig. 10). Notably, brain bacterial loads increased with time regardless of mouse genotype or treatment (equivalent to a 10,000-fold increase between 6 h postinfection and presentation of clinical disease), indicating rapid and efficient replication of S. suis within the CNS.

**FIG 11:**
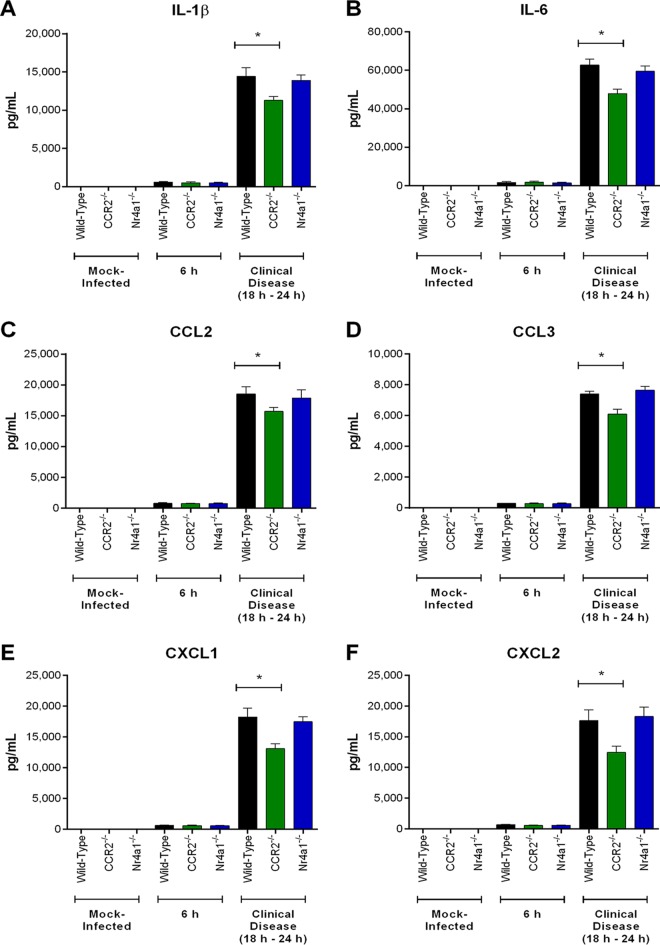
Inflammatory but not patrolling monocytes contribute to S. suis-induced central nervous system (CNS) inflammation. Brain levels of IL-1β (A), IL-6 (B), CCL2 (C), CCL3 (D), CXCL1 (E), and CXCL2 (F) in wild-type, CCR2^−/−^, and Nr4a1^−/−^ mice following intracisternal mock infection (THB) or 6 h following infection with 10^5^ CFU of S. suis or upon presentation of clinical CNS disease. The data represent the mean ± SEM (*n* = 5). * (*P* < 0.05) indicates a significant difference in mediator levels between wild-type and CCR2^−/−^ mice as determined using the unpaired *t* test.

**FIG 12:**
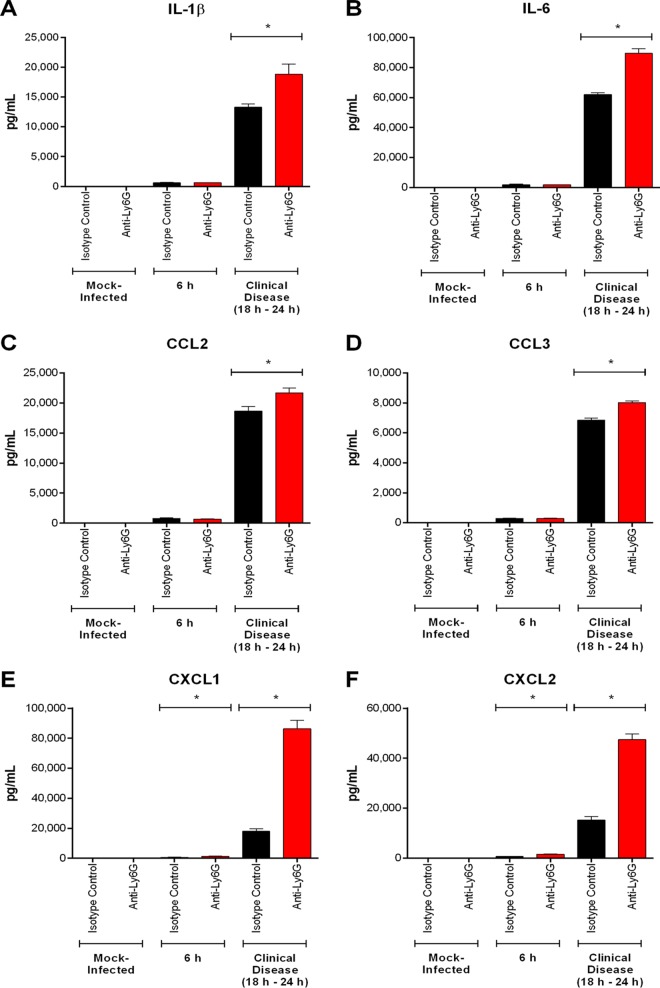
The presence of neutrophils modulates S. suis-induced central nervous system (CNS) inflammation. Brain levels of IL-1β (A), IL-6 (B), CCL2 (C), CCL3 (D), CXCL1 (E), and CXCL2 (F) in wild-type mice pretreated with either isotype control or anti-Ly6G neutralizing antibody mice following intracisternal mock infection or 6 h following infection with 10^5^ CFU of S. suis or upon presentation of clinical CNS disease. The data represent the mean ± SEM (*n* = 5). * (*P* < 0.05) indicates a significant difference in mediator levels between isotype control- and anti-Ly6G-treated mice as determined using the unpaired *t* test.

**FIG 13:**
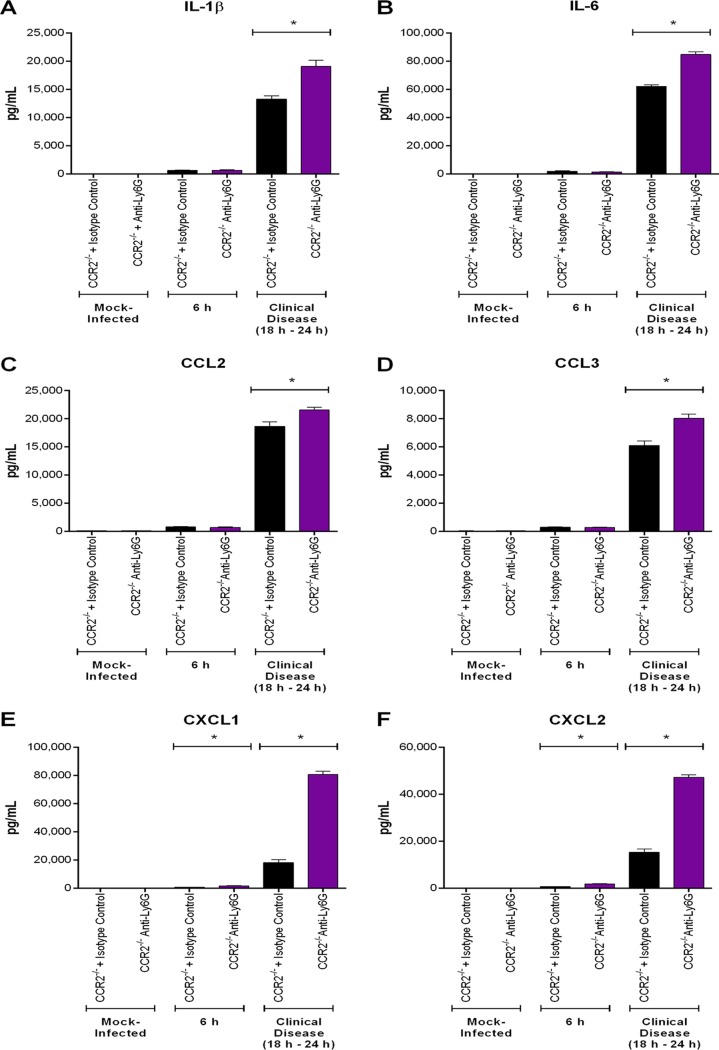
Neutrophils modulate S. suis-induced central nervous system (CNS) inflammation even in the absence of inflammatory monocytes. Brain levels of IL-1β (A), IL-6 (B), CCL2 (C), CCL3 (D), CXCL1 (E), and CXCL2 (F) in CCR2^−/−^ mice pretreated with either isotype control or anti-Ly6G neutralizing antibody mice following intracisternal mock infection or 6 h following infection with 10^5^ CFU of S. suis or upon presentation of clinical CNS disease. The data represent the mean ± SEM (*n* = 5). * (*P* < 0.05) indicates a significant difference in mediator levels between isotype control- and anti-Ly6G-treated mice as determined using the unpaired *t* test.

Since the presence of S. suis in the CNS induces local inflammation (4), brain proinflammatory mediators were measured 6 h postinfection and upon presentation of clinical CNS disease (between 18 h and 24 h postinfection). Levels of IL-1β, IL-6, CCL2, CCL3, CXCL1, and CXCL2 were significantly greater 6 h following infection than in mock-infected mice (*P* < 0.05) but were similar among wild-type, CCR2^−/−^, and Nr4a1^−/−^ mice (Fig. 11). Upon presentation of clinical CNS disease, however, they were significantly lower in the CNS of CCR2^−/−^ mice than in their wild-type and Nr4a1^−/−^ counterparts (*P* < 0.05), which presented similar levels (Fig. 11). It is worth noting that the reduction in CCR2^−/−^ mice was equivalent to a 25% decrease at most (Fig. 11). In contrast, CXCL1 and CXCL2 production was significantly increased 6 h postinfection in the CNS of neutrophil-depleted mice (*P* < 0.05) (Fig. 12). Upon presentation of clinical CNS disease, however, levels of IL-1β, IL-6, CCL2, CCL3, CXCL1, and CXCL2 were significantly higher following anti-Ly6G treatment than in isotype control-treated mice, with a 300% increase in CXCL1 and CXCL2 production (*P* < 0.05) (Fig. 12). Interestingly, anti-Ly6G treatment of CCR2^−/−^ mice resulted in an increase in CNS proinflammatory mediator production (*P* < 0.05) similar to that in anti-Ly6G-treated wild-type mice (neutrophil-depleted only) (Fig. 13).

This differential role of inflammatory monocytes and neutrophils in S. suis-induced CNS disease was observed using a standard dose of S. suis. Since the initial presence of elevated bacterial burdens might affect the role or outcome observed, the minimal bacterial dose capable of inducing CNS disease (10 CFU) was inoculated via the intracisternal route (9). Wild-type, CCR2^−/−^, or Nr4a1^−/−^ mice and isotype control- or anti-Ly6G-treated wild-type or CCR2^−/−^ mice all developed similar clinical CNS disease, with 50% to 60% of mice presenting clinical signs between 36 h and 72 h postinfection (Fig. S5). Moreover, similar patterns in brain bacterial loads, both early (24 h postinfection) and upon presentation of clinical CNS disease (between 36 h and 72 h postinfection), were observed as when using the standard dose (Fig. S6). Notably, only certain mice presented brain bacterial burden 24 h postinfection, corresponding to the mice that eventually developed clinical disease, while no burden was detected in the CNS of mice that never presented clinical disease by the end of the experiment (72 h postinfection) (Fig. S6). Finally, patterns of IL-1β, IL-6, CCL2, CCL3, CXCL1, and CXCL2 production in the CNS of mice were also similar to the standard dose (Fig. S7 to S9). Taken together, these results demonstrate that while inflammatory monocytes and neutrophils have differing roles during S. suis-induced CNS disease, their presence is not critical for the development of clinical CNS disease.

## DISCUSSION

Diseases caused by S. suis serotype 2 are the consequence of elevated bacterial replication usually leading to exacerbated systemic and CNS inflammatory responses. While monocytes and neutrophils are the predominant innate immune peripheral blood cells, their role during the S. suis-induced systemic infection remains unknown. Moreover, meningitis is a consequence of excessive local inflammation and is characterized by the infiltration of monocytes and neutrophils (3, 8, 9). However, it is unknown whether these cells are a cause or a consequence of this local inflammatory response.

Once in the bloodstream, S. suis can replicate and disseminate, resulting in innate host cell activation and induction of an inflammatory response (3, 4, 37). Results obtained herein demonstrate that inflammatory monocytes and neutrophils actively participate in S. suis-induced systemic inflammation, since all proinflammatory mediators evaluated were reduced in their absence. This agrees with *in vitro* studies using human THP-1 monocytes, from which S. suis induced the secretion of tumor necrosis factor (TNF) and CCL2 (24). Furthermore, this is the first study to report a role of monocytes and neutrophils in proinflammatory mediator production during S. suis infection *in vivo*. In fact, to our knowledge, no other study has ever evaluated proinflammatory mediator production by neutrophils.

In addition, the absence of neutrophils and inflammatory monocytes resulted in increased blood bacterial burdens and, consequently, decreased mouse survival. Since a certain level of inflammation is required for bacterial clearance (10), this indicates that their contribution to systemic inflammation is necessary for host outcome during S. suis infection. In addition to this indirect role in bacterial clearance, neutrophils also appear to be directly involved in early elimination of S. suis from the bloodstream. Indeed, blood bacterial burden was already greater in neutrophil-depleted mice 6 h postinfection, at which time phagocytic and killing mechanisms induced by inflammation have probably not been optimally activated since S. suis-induced inflammation peaks around 12 h postinfection (35). This supports *in vitro* studies showing that neutrophils are more efficient at killing S. suis than monocytes without prior priming (22, 23).

Overall, our results indicate that neutrophils and inflammatory monocytes play a beneficial role during S. suis systemic infection. Likewise, inflammatory monocytes play a beneficial role during group A *Streptococcus* infection by participating in systemic bacterial elimination via as yet unknown mechanisms (38). Moreover, neutrophils play a crucial role during GBS, S. pneumoniae, and Staphylococcus aureus infection via direct (killing) and/or indirect (inflammation) bacterial control (33, 39, 40). Though induced inflammation is required for bacterial clearance, its exacerbation is detrimental to the host and causes death (3, 4, 37). While associated with a “beneficial role,” participation of inflammatory monocytes and neutrophils in the exaggerated inflammation that usually leads to death in wild-type infected animals cannot be ruled out. Similar results were observed in the absence of MyD88 signaling, which is critical for S. suis-induced inflammation (9). This indicates that the systemic inflammatory response is precariously balanced: too little inflammation results in uncontrolled bacterial replication on the one hand (10), while exacerbated inflammation causes tissue damage and organ failure on the other (3, 35). In the present study, CCR2^−/−^ and neutrophil-depleted mice clearly presented higher mortality not necessarily due to exaggerated inflammation. As such, mice probably died from tissue and organ damage directly caused by the uncontrolled levels of systemic bacteria (9). Indeed, S. suis possesses numerous cytotoxic factors, including the toxin suilysin, that may cause organ failure (41).

Unlike inflammatory monocytes, no significant contribution of patrolling monocytes to the S. suis-induced systemic infection was observed. Because they are traditionally associated with patrolling, tissue repair, and homeostatic functions (19, 42), this was not entirely surprising. However, knowledge of their role in bacterial infections is very limited. In fact, this is one of only a few studies investigating their role. It was reported, however, that patrolling monocytes support Porphyromonas gingivalis survival and infection-driven bone resorption by hindering neutrophil infiltration and bacterial clearance (43).

Following systemic infection, surviving individuals are susceptible to developing a life-threatening CNS disease (3, 4), during which infiltration of monocytes and neutrophils has been well documented (2, 3, 8, 21). S. suis-induced meningitis is classified as suppurative, indicating a predominance of neutrophils, which concurs with results obtained in this study, accompanied by inflammatory, but few patrolling, monocytes. Similar results were also observed during S. pneumoniae and E. coli meningitis, possibly suggesting a commonality of bacterial meningitis (31, 32). Interestingly, inflammatory (but not patrolling) monocytes were partially implicated in S. suis-induced CNS inflammation, but not in brain bacterial burden control, with no influence on development of clinical CNS disease. Similarly, inflammatory monocytes were not involved in S. pneumoniae meningitis, while their absence resulted in higher mortality during E. coli K1 meningitis (31, 32). On the other hand, and differently from what was reported for S. pneumoniae (31), neutrophils were involved in the control and elimination of S. suis from the CNS, but not in CNS inflammation. In fact, their depletion increased proinflammatory mediator production as previously reported for E. coli K1 (32). While surprising, this increase could result from the increased brain bacterial burden in their absence further activating resident immune cells, resulting in inflammatory mediator amplification (32).

The surprising development of clinical CNS disease in the absence of inflammatory monocytes or neutrophils suggested a possible compensatory effect. In fact, resident CNS cells, probably microglia and astrocytes, would be mainly responsible for not only the local inflammation but also clinical disease. Resident CNS cells have not only been previously shown to produce IL-6, CCL2, and CXCL1 following S. suis infection *in vitro* (21, 44, 45), but they are also associated with CCL2 expression *in vivo* (3). Since it has been previously reported that depletion of both inflammatory monocytes and neutrophils resulted in a nearly complete absence of clinical CNS disease following S. pneumoniae or E. coli infection (31, 32), the present study is the first to report no critical role of either infiltrating inflammatory monocytes or neutrophils in the development of bacterial meningitis. Indeed, infiltration of these cells into the CNS during S. suis infection would be a consequence and not a cause of the extremely high levels of chemokines produced within. A noncritical role of infiltrating monocytes and neutrophils was also observed in a model of cuprizone-induced demyelination (46). Furthermore, infiltrating inflammatory monocytes may contribute not only to their own recruitment, given the decrease in CCL2 production in their absence (a key chemokine involved in inflammatory monocyte recruitment) (19), but also to that of neutrophils, via CCL3, CXCL1, and CXCL2 production. Indeed, CCL3 has been previously reported to participate in the recruitment of neutrophils to the CNS during Haemophilus influenzae type b and S. pneumoniae infections (47, 48), whereas CXCL1 and CXCL2 are potent neutrophil chemoattractants (49). Accordingly, a lack of CXCL2 reduces recruitment of neutrophils to the CNS during H. influenzae type b meningitis (47).

In conclusion, inflammatory monocytes and neutrophils participate in the inflammatory response required for clearance of S. suis during systemic infection. In contrast, they partially contribute to S. suis-induced CNS inflammation and bacterial elimination, respectively. However, their overall role in clinical CNS disease is redundant. This indicates that even though they infiltrate into the CNS because of the elevated chemokine production, resident immune cells are mostly responsible for S. suis-induced CNS inflammation and clinical disease, with inflammatory monocytes and neutrophils contributing to the inflammatory amplification loop. Consequently, further studies targeting resident CNS immune cells will be necessary to better understand their role and the underlying mechanism involved.

## MATERIALS AND METHODS

### Ethics statement.

This study was carried out in accordance with the recommendations of the guidelines and policies of the Canadian Council on Animal Care and the principles set forth in the *Guide for the Care and Use of Laboratory Animals* (58). The protocols and procedures were approved by the Animal Welfare Committee of the University of Montreal (protocol number Rech-1570).

### Mice.

CCR2^−/−^ (B6.129S4-*Ccr2^tm1lfc^*/J) and Nr4a1^−/−^ (B6.129S2-*Nr4a1^tm1Jmi^*/J) mice on C57BL/6J background were purchased from Jackson Research Laboratories (Bar Harbor, ME, USA) and housed under specific-pathogen-free conditions alongside their C57BL/6J wild-type counterparts. CCR2 is required for the egress of inflammatory monocytes from the bone marrow into the bloodstream and for their migration into infected or damaged tissues in a pathology-dependent manner following expression of CCL2 (28, 46, 50–52). Consequently, CCR2^−/−^ mice have significantly reduced numbers of inflammatory monocytes in blood due to their retention within the bone marrow (28, 46, 50–52). Meanwhile, the orphan nuclear transcription factor Nr4a1 is required for the differentiation and survival of patrolling monocytes, resulting in Nr4a1^−/−^ mice having a deficiency in patrolling monocytes (29). Given these phenotypes, CCR2^−/−^ and Nr4a1^−/−^ mice were used in this study to evaluate the role of these two monocyte subsets in S. suis-induced systemic and CNS diseases.

### S. suis strain and growth conditions.

The well-characterized and highly encapsulated classical virulent S. suis serotype 2 P1/7 strain, isolated from pig with meningitis in the United Kingdom, was used throughout this study (53). S. suis was grown overnight on Columbia agar supplemented with 5% sheep blood (Oxoid, Nepean, ON, Canada) at 37°C with 5% CO_2_. Then, 5 ml of Todd Hewitt broth (THB; Becton, Dickinson, Mississauga, ON, Canada) was inoculated with an isolated colony and incubated for 8 h at 37°C with 5% CO_2_. Working cultures were prepared by inoculating 30 ml of THB with 10 μl of a 10^−3^ dilution of the 8-h culture and incubating it for 16 h at 37°C with 5% CO_2_. Bacteria were washed twice with phosphate-buffered saline (PBS; pH 7.3) and resuspended in THB, and the final CFU/ml was determined by plating on THB agar (THA).

### Neutrophil depletion.

Mice (wild-type and CCR2^−/−^) were intraperitoneally injected with 0.5 mg of rat monoclonal anti-mouse Ly6G antibody (clone 1A8) or rat IgG2a isotype control (clone RTK2758) (BioLegend, Burlington, ON, Canada) 24 h prior to infection with S. suis as previously described for other pathogens (33, 54, 55). The 1A8 anti-Ly6G antibody was used for its specificity and depletion efficiency (56). To confirm depletion, 50 μl of peripheral blood was collected at different times following injection, anticoagulated with EDTA, and treated with an FcR-blocking reagent (FcγIII/II Rc Ab; BD Pharmingen, Mississauga, ON, Canada) for 15 min on ice. Cells were stained with fluorescein isothiocyanate (FITC)-conjugated anti-Ly6G (clone 1A8) or isotype antibody (BD Pharmingen) as a control for 45 min on ice, and erythrocytes were lysed using a 155-mM NH_4_Cl, 12-mM NaHCO_3_, and 0.1-mM EDTA solution. Samples were then analyzed on the BD FACSAria Fusion instrument using the FACSDiva software (BD Biosciences, Mississauga, ON, Canada). Ly6G^+^ cells were depleted beyond 85% (Table S1; Fig. S1).

### S. suis experimental mouse infections.

Six-week-old male and female mice were used throughout this study. Mice were acclimatized to standard laboratory conditions with unlimited access to water and rodent chow (4, 35). These studies were carried out in strict accordance with the recommendations of and approved by the University of Montreal Animal Welfare Committee guidelines and policies, including euthanasia to minimize animal suffering, applied throughout this study when animals were seriously affected since mortality was not an endpoint measurement. For systemic virulence studies, 1 × 10^7^ CFU of S. suis was administered by intraperitoneal inoculation to mice for survival and blood bacterial burden. Mice were monitored at least thrice daily until 72 h postinfection for clinical signs of systemic disease (rough coat hair, closed/swollen eyes, prostration, depression, difficulty breathing, and lethargy). The blood bacterial burden of the surviving mice was assessed at different times postinfection by collecting 5 μl of blood from the caudal tail vein and appropriately diluting and plating it on THA as described above. Blood bacterial burden was also measured prior to euthanasia.

For the transcutaneal intracisternal model of CNS infection, mice were anesthetized with inhaled isoflurane (Pharmaceutical Partners of Canada, Richmond Hill, ON, Canada), and 10 μl of 1 × 10^3^ CFU/ml or 1 × 10^7^ CFU/ml (final concentrations of 10 CFU and 10^5^ CFU, respectively) of S. suis was injected as previously described (4, 36). Animals were monitored every 8 h until 72 h postinfection and euthanized at different predetermined time points upon presentation of clinical signs of CNS disease (spatial disorientation, hyper-excitement followed by opisthotonos, circular walking with head tilt, sudden spinning while in recumbence, and tonicoclonic movements), as required by the Animal Welfare Committee of the University of Montreal for ethical reasons, or at the end of the study. Controls (mock-infected) were injected with 10 μl of the vehicle solution (sterile THB).

### Measurement of plasma (systemic) proinflammatory mediator levels.

Additional groups of mice were intraperitoneally infected with 1 × 10^7^ CFU of S. suis as described above. Mice were euthanized 12 h postinfection (4, 37), and blood was collected by intracardiac puncture and anticoagulated with EDTA (Sigma-Aldrich, Oakville, ON, Canada). Plasma was collected following centrifugation at 10,000 × *g* for 10 min at 4°C and stored at –80°C. Plasmatic concentrations of interleukin-6 (IL-6), IL-12p70, interferon-γ (IFN-γ), C-C motif chemokine ligand 3 (CCL3), CCL4, CCL5, C-X-C motif chemokine ligand 1 (CXCL1), and CXCL2 were measured using a custom-made cytokine Bio-Plex Pro assay (Bio-Rad, Hercules, CA, USA) according to the manufacturer’s instructions. Acquisition was performed on the MAGPIX platform (Luminex), and data were analyzed using Bio-Plex Manager 6.1 software (Bio-Rad).

### Measurement of brain bacterial load and proinflammatory mediator levels.

Additional groups of mice were intracisternally infected with 10 CFU or 10^5^ CFU, and samples were collected at different predetermined time points, upon presentation of clinical signs of CNS disease, or at the end of the experiment (24 h or 72 h postinfection following infection with 10^5^ CFU and 10 CFU, respectively). Following euthanasia, the brains were aseptically recovered and homogenized in PBS from which bacterial burdens were determined by plating appropriate dilutions on THA as described above or were directly frozen in liquid nitrogen. For proinflammatory mediator evaluation, extraction buffer, prepared using cOmplete Mini EDTA-free protease inhibitor cocktail tablets (Roche Diagnostics GmbH, Mannheim, Germany) according to the manufacturer’s instructions and supplemented with 0.4% CHAPS (Sigma-Aldrich), was added to frozen brains, which were then homogenized using a Polytron PT 1200E system bundle (Kinematica, Lucerne, Switzerland). Brain homogenate supernatants were collected following centrifugation at 10,000 × *g* for 10 min at 4°C and stored at –80°C. Levels of IL-6, CCL2, CCL3, and CXCL2 were measured using a custom-made cytokine Bio-Plex Pro assay (Bio-Rad) as described above, while levels of IL-1β and CXCL1 were measured with sandwich enzyme-linked immunosorbent assay using pair-matched antibodies (R&D Systems, Minneapolis, MN, USA) as previously described (12).

### Evaluation of central nervous system blood monocyte and neutrophil infiltrates.

Upon presentation of clinical signs of CNS disease, mice were euthanized and immediately perfused with PBS to remove blood leukocytes. The brains were then recovered and cells isolated as previously described, including use of a Percoll gradient (57). Cells were treated with an FcR-blocking reagent (BD Pharmingen) for 15 min and stained with BV421-conjugated CD11b (clone M1/70), APC/Cy7-conjugated CD45.2 (clone 104), APC-conjugated-Ly6C (clone AL21), and FITC-conjugated anti-Ly6G (clone 1A8) or isotype antibodies (all from BD Pharmingen) as a control for 45 min on ice. Samples were then analyzed on the BD FACSAria Fusion instrument using the FACSDiva software (BD Biosciences).

### Brain histopathological studies.

Upon presentation of clinical signs of CNS disease or at the end of the study, animals were euthanized and brains recovered and fixed in 10% buffered formalin. After paraffin embedding, 4-μm-thick sections of the brain were stained with hematoxylin phloxine saffron following the standard protocol and examined under light microscopy. Brains were qualitatively examined for the presence or absence of histopathological lesions of S. suis-induced CNS disease (massive suppuration, leukocyte infiltration, multifocal gliosis, hemorrhages, and necrosis).

### Statistical analyses.

For all data, the normality of distribution was verified using the Shapiro-Wilk test. Accordingly, parametric tests (unpaired *t* test) or nonparametric tests (Mann-Whitney rank sum test) were performed to evaluate statistical differences between groups. Data are presented as the mean ± standard error of the mean (SEM) or as the geometric mean. Log-rank (Mantel-Cox) tests were used to compare survival between groups of mice. *P* < 0.05 was considered statistically significant.

## Supplementary Material

Supplemental file 1
