# Supplementary material for: Inflammatory Monocytes and Neutrophils Regulate Streptococcus suis-Induced Systemic Inflammation and Disease but Are Not Critical for the Development of Central Nervous System Disease in a Mouse Model of Infection
Source: Infect Immun. 2020 Feb 20;88(3):e00787-19. doi: 10.1128/IAI.00787-19 (PMC7035915; doi:10.1128/IAI.00787-19)
Supplement: Supplemental file 1 [file zii999093003s1.pdf]

Table S1. Percentage of neutrophils in peripheral blood following treatment with anti-Ly6G neutralizing antibody or its isotype control.

Treatment	Time post-injection (h)		
	**24**	**48**	**72**
Isotype Control	7.8 ± 0.2	7.2 ± 0.6	7.6 ± 0.4
Anti-Ly6G	0.8 ± 0.3*	0.9 ± 0.4*	1.0 ± 0.3*

Data represent mean ± SEM (n = 3). * ($p < 0.05$) indicates a significant difference between groups as determined using the Mann-Whitney rank sum test.

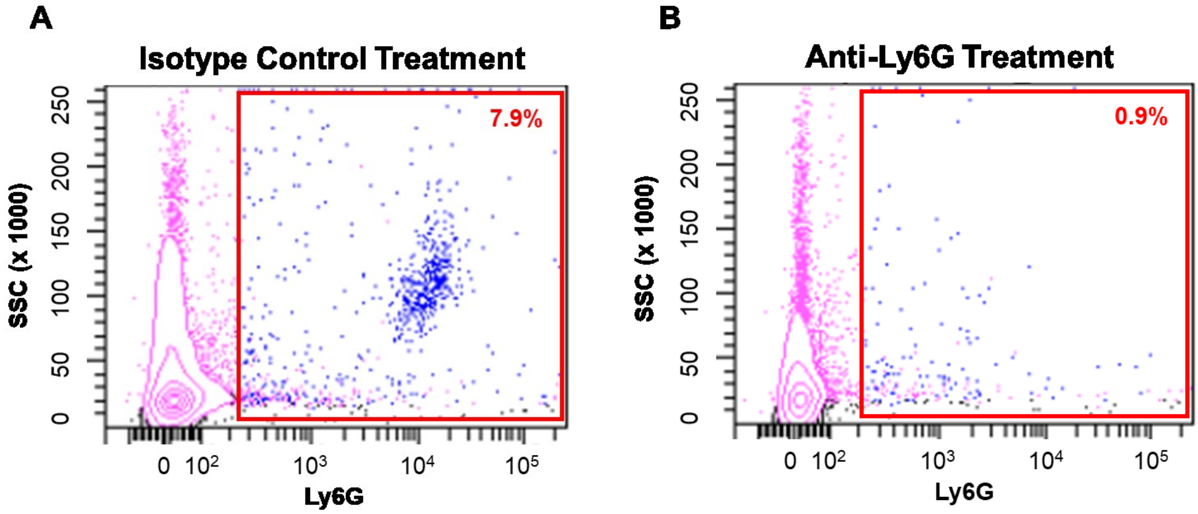

Fig. S1. Efficiency of *in vivo* neutrophil depletion. Wild-type mice were pre-treated with either isotype control (mock-treated) (**A**) or anti-Ly6G monoclonal neutralizing antibody (**B**) and peripheral blood was collected 48 h following injection. Cells were stained with FITC-conjugated anti-Ly6G. Representative dot plots showing neutrophil percentages in mock-treated or anti-Ly6G-treated mice as analyzed by flow cytometry.

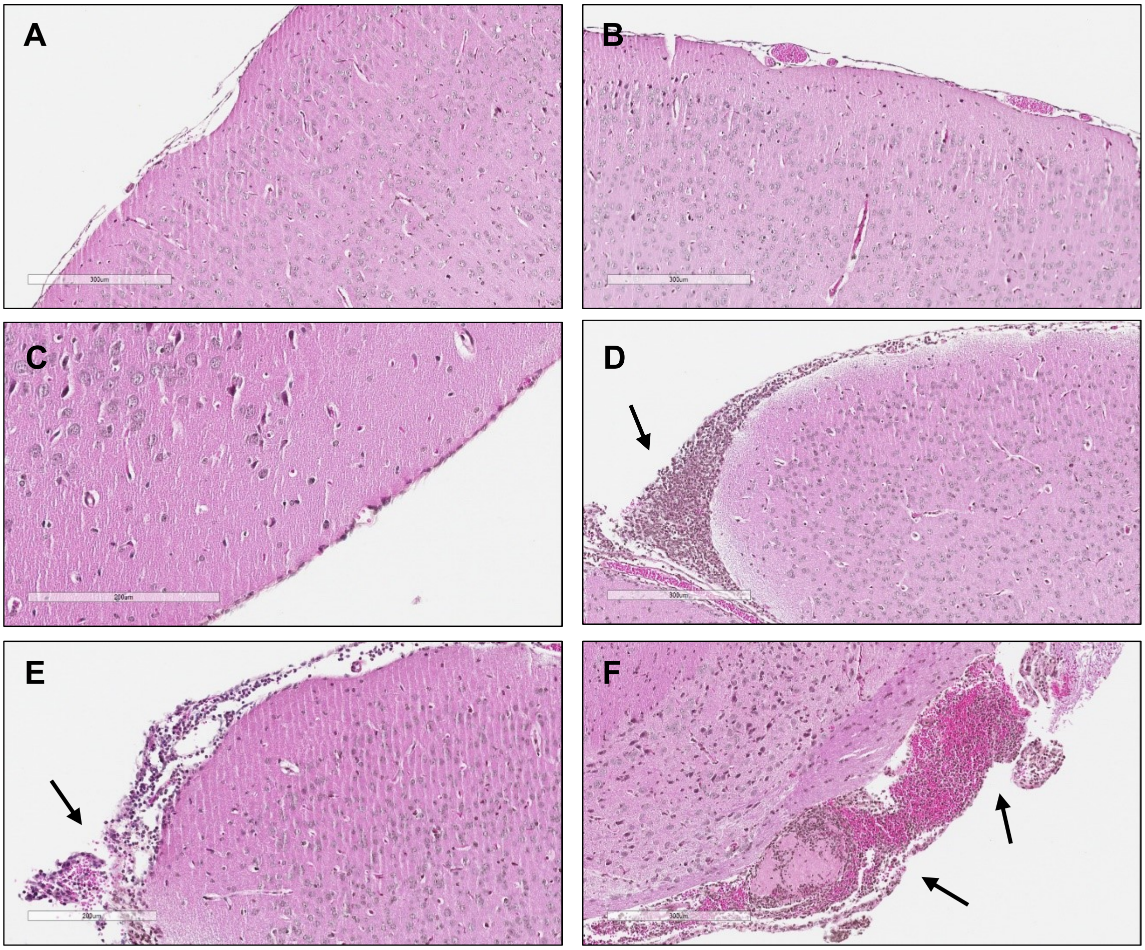

Fig. S2. Histopathological lesions of *S. suis*-induced central nervous system (CNS) disease in the absence of inflammatory or patrolling monocytes. Representative micrographs of the brain of mock-infected wild-type (**A**), CCR2$^{-/-}$ (**B**) and Nr4a1$^{-/-}$ (**C**) mice [absence of histopathological lesions in **A**, **B** and **C**] or wild-type [suppuration and leukocyte infiltration] (**D**), CCR2$^{-/-}$ [suppuration and leukocyte infiltration] (**E**) and Nr4a1$^{-/-}$ [suppuration, leukocyte infiltration and hemorrhaging] (**F**) mice infected with a standard dose of *S. suis* (1 x 10^5 CFU) by intracisternal inoculation. Brains were collected upon presentation of clinical CNS disease or at the end of the experiment (24 h post-infection). Black arrowheads indicate lesions of *S. suis*-induced CNS disease.

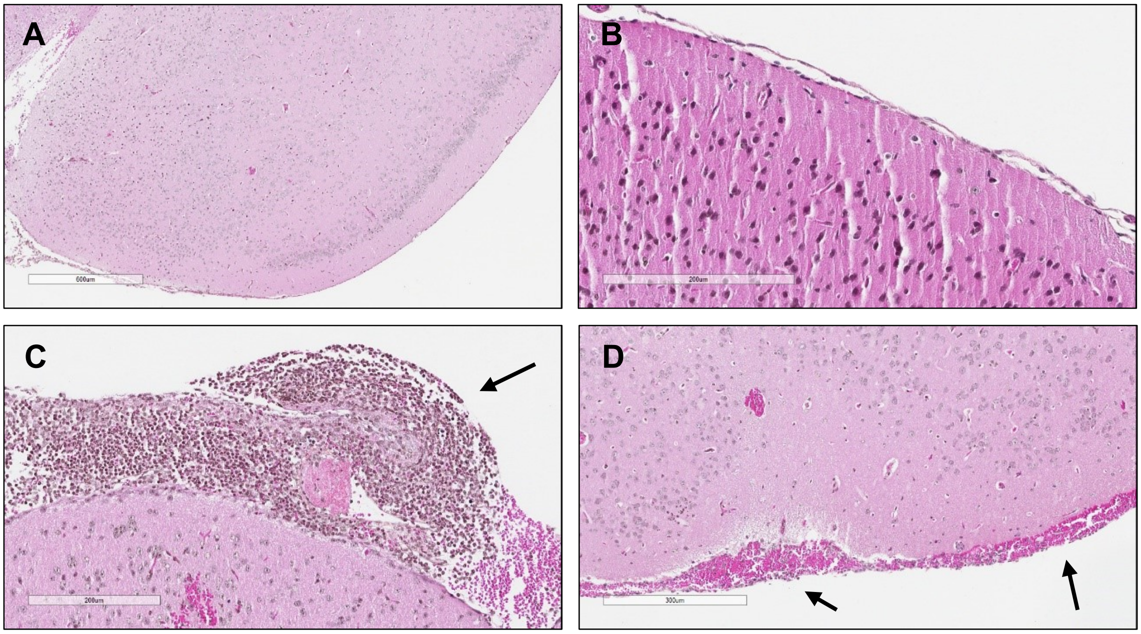

Fig. S3. Histopathological lesions of *S. suis*-induced central nervous system (CNS) disease following neutrophil depletion. Representative micrographs of the brain of mock-infected wild-type mice pre-treated with either isotype control (**A**) or anti-Ly6G neutralizing antibody (**B**) [absence of histopathological lesions in **A** and **B**] or wild-type mice pre-treated with either isotype control [suppuration and leukocyte infiltration] (**C**) or anti-Ly6G neutralizing antibody [leukocyte infiltration and hemorrhaging] (**D**) mice infected with a standard dose of *S. suis* (1×10^5 CFU) by intracisternal inoculation. Brains were collected upon presentation of clinical CNS disease or at the end of the experiment (24 h post-infection). Black arrowheads indicate lesions typical of *S. suis*-induced CNS disease.

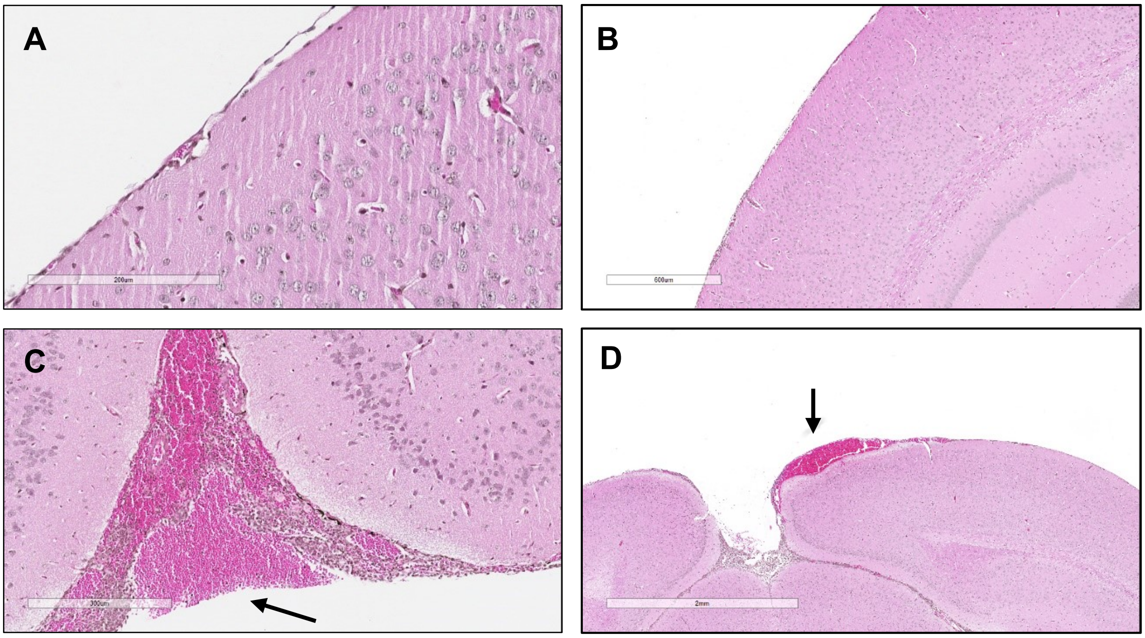

Fig. S4. Histopathological lesions of *S. suis*-induced central nervous system (CNS) disease following neutrophil depletion in the absence of inflammatory monocytes. Representative micrographs of the brain of mock-infected CCR2$^{-/-}$ mice pre-treated with either isotype control (**A**) or anti-Ly6G neutralizing antibody (**B**) [absence of histopathological lesions in **A** and **B**] or CCR2$^{-/-}$ mice pre-treated with either isotype control [suppuration and leukocyte infiltration] (**C**) or anti-Ly6G neutralizing antibody [leukocyte infiltration and hemorrhaging] (**D**) infected with a standard dose of *S. suis* (1 x 10^5 CFU) by intracisternal inoculation. Brains were collected upon presentation of clinical CNS disease or at the end of the experiment (24 h post-infection). Black arrowheads indicate lesions typical of *S. suis*-induced CNS disease.

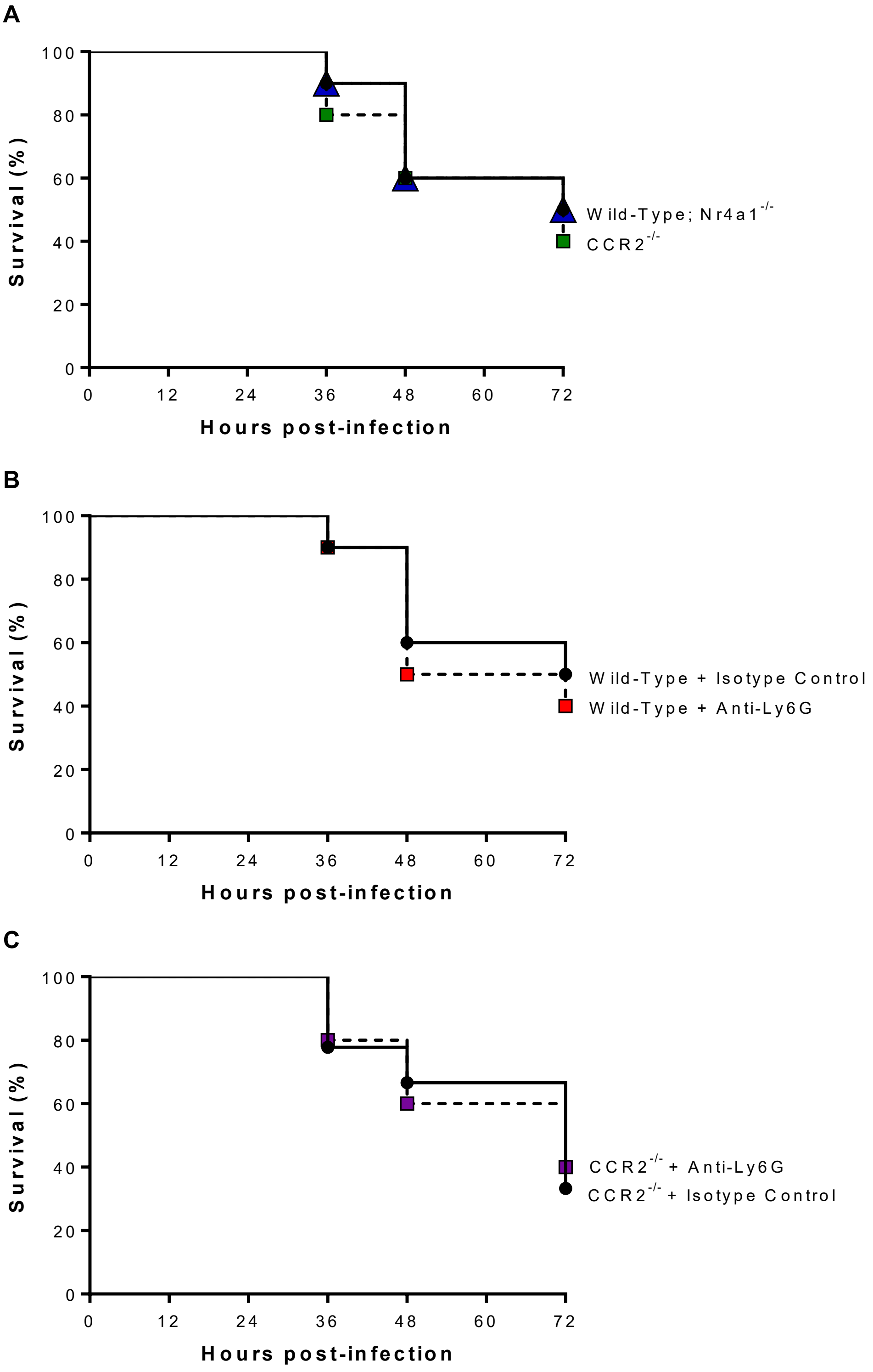

Fig. S5. Monocytes and neutrophils are not required for the development of clinical central nervous system (CNS) disease following infection with a low dose of *S. suis*. Survival of wild-type (black), CCR2$^{-/-}$ (green) or Nr4a1$^{-/-}$ (blue) mice (**A**), wild-type mice pre-treated with either isotype control (black) or anti-Ly6G neutralizing antibody (red) (**B**) or CCR2$^{-/-}$ mice pre-treated with either isotype control (black) or anti-Ly6G neutralizing antibody (purple) (**C**) following intracisternal infection with 10 CFU of *S. suis*. Data represent survival curves of mice euthanized upon presentation of clinical signs of CNS disease (n = 10). Statistical differences were analyzed using the Log-rank (Mantel-Cox) test.

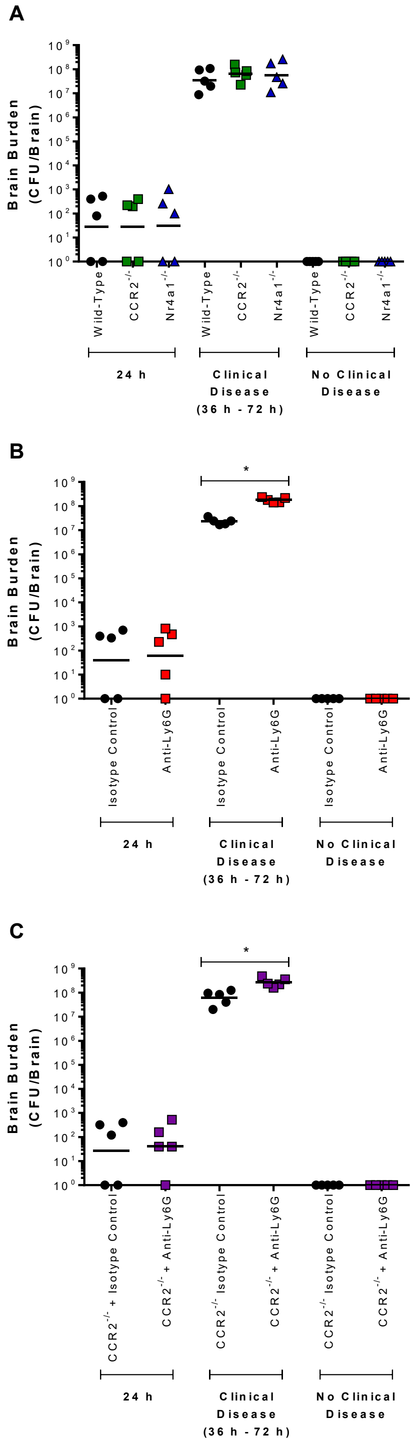

Fig. S6. Neutrophils but not monocytes participate in brain bacterial burden control following infection with a low dose of *S. suis*. Brain bacterial burden of wild-type, CCR2$^{-/-}$ or Nr4a1$^{-/-}$ mice, wild-type mice pre-treated with either isotype control or anti-Ly6G neutralizing antibody or CCR2$^{-/-}$ mice pre-treated with either isotype control or anti-Ly6G neutralizing antibody 24 h following intracisternal infection with 10 CFU of *S. suis*, upon presentation of clinical central nervous system disease or at the end of the infection (no clinical disease). Data represent geometric mean (n = 5). * ($p < 0.05$) indicates a significant difference between blood bacterial burden of isotype control- and anti-Ly6G-treated mice (wild-type or CCR2$^{-/-}$) as determined using the Mann-Whitney rank sum test.

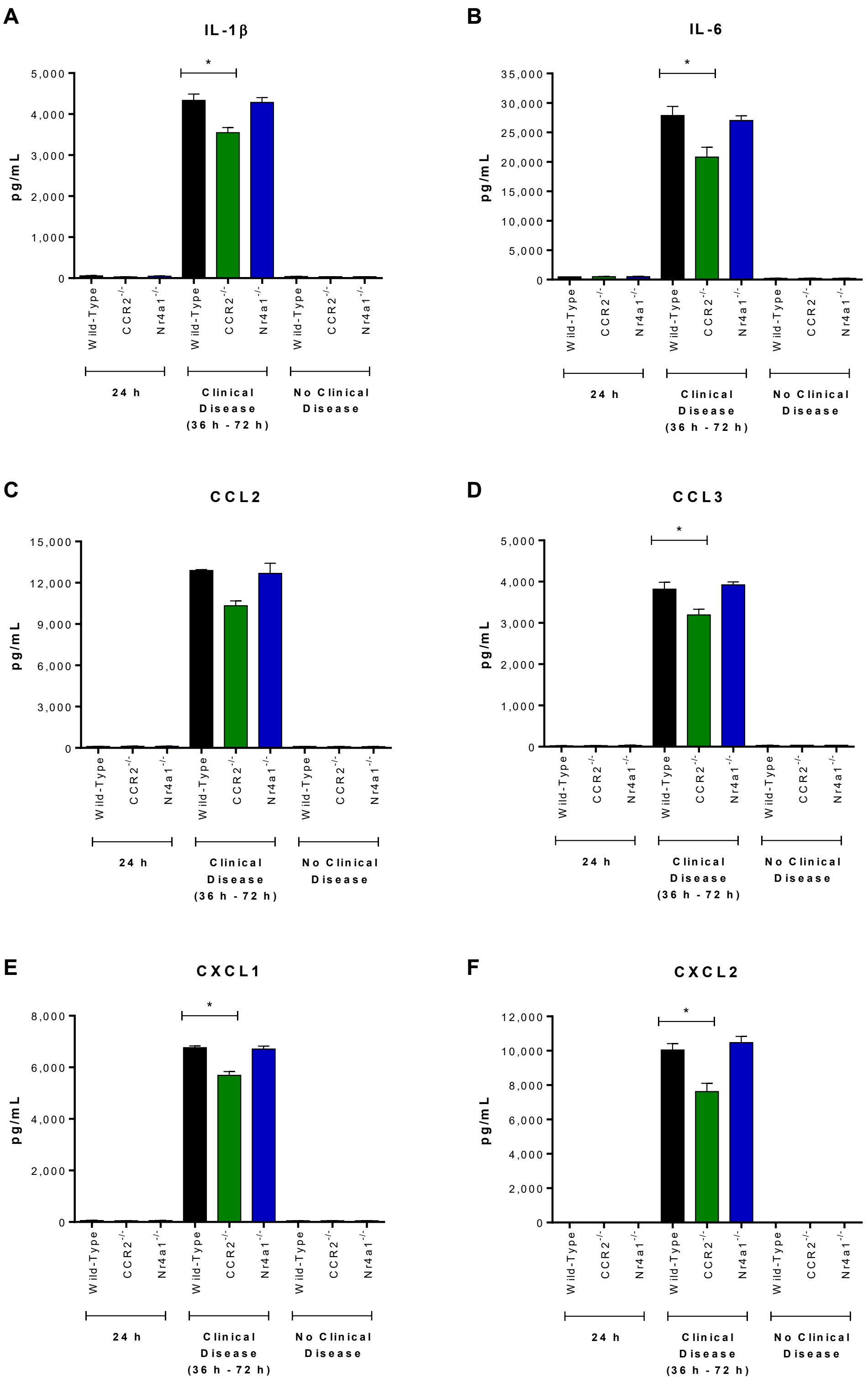

Fig. S7. Inflammatory but not patrolling monocytes contribute to *S. suis*-induced central nervous system (CNS) inflammation following infection with a low bacterial dose. Brain levels of IL-1β (**A**), IL-6 (**B**), CCL2 (**C**), CCL3 (**D**), CXCL1 (**E**), and CXCL2 (**F**) in wild-type, CCR2$^{-/-}$ or Nr4a1$^{-/-}$ mice 24 h following intracisternal infection with 10 CFU of *S. suis*, upon presentation of clinical CNS disease or at the end of the infection (no clinical disease). Data represent mean ± SEM (n = 5). * ($p < 0.05$) indicates a significant difference in mediator levels between wild-type and CCR2$^{-/-}$ mice as determined using the unpaired t-test.

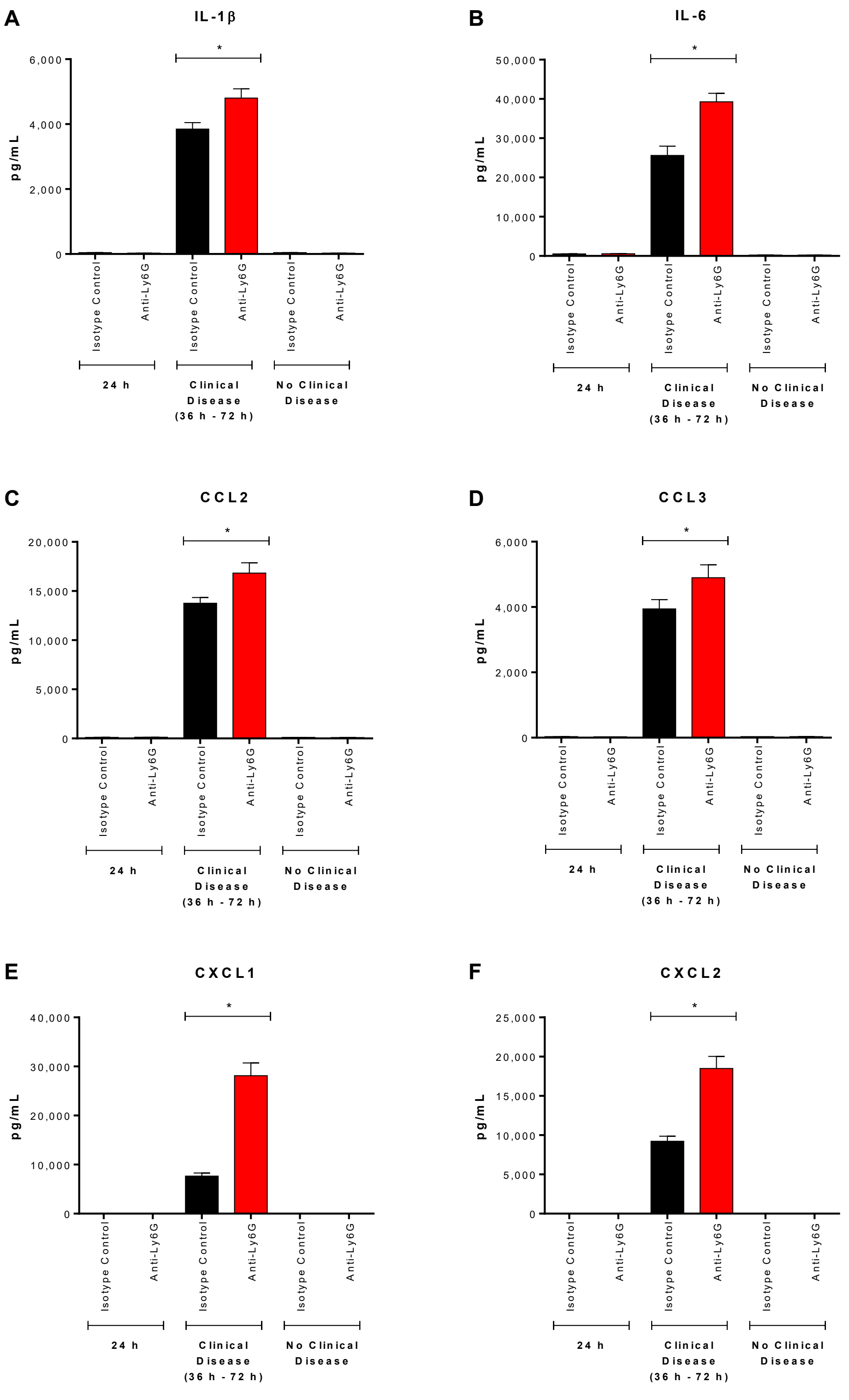

Fig. S8. Presence of neutrophils modulates *S. suis*-induced central nervous system (CNS) inflammation following infection with a low bacterial dose. Brain levels of IL-1β (**A**), IL-6 (**B**), CCL2 (**C**), CCL3 (**D**), CXCL1 (**E**), and CXCL2 (**F**) in wild-type mice pre-treated with either isotype control or anti-Ly6G neutralizing antibody 24 h following intracisternal infection with 10 CFU of *S. suis*, upon presentation of clinical CNS disease or at the end of the infection (no clinical disease). Data represent mean ± SEM (n = 5). * ($p < 0.05$) indicates a significant difference in mediator levels between isotype control- and anti-Ly6G-treated mice as determined using the unpaired t-test.

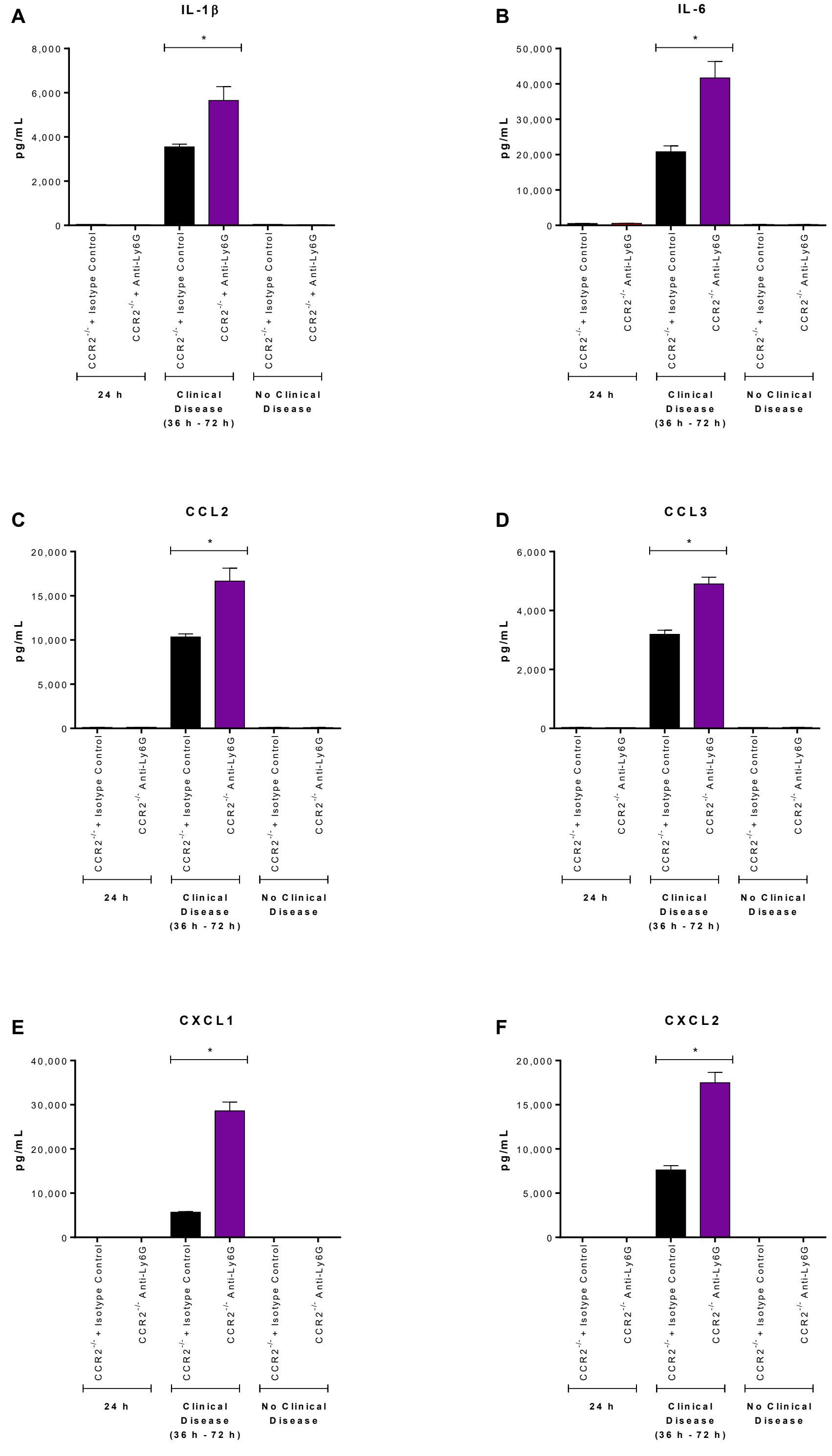

Fig. S9. Neutrophils modulate *S. suis*-induced central nervous system (CNS) inflammation even in the absence of inflammatory monocytes following infection with a low bacterial dose. Brain levels of IL-1β (**A**), IL-6 (**B**), CCL2 (**C**), CCL3 (**D**), CXCL1 (**E**), and CXCL2 (**F**) in CCR2$^{-/-}$ mice pre-treated with either isotype control or anti-Ly6G neutralizing antibody mice following intracisternal mock-infection or 6 h following infection with 10 CFU of *S. suis* or upon presentation of clinical CNS disease. Data represent mean ± SEM (n = 5). * ($p < 0.05$) indicates a significant difference in mediator levels between isotype control- and anti-Ly6G-treated mice as determined using the unpaired t-test.
